# Polyphyllin D Shows Anticancer Effect through a Selective Inhibition of Src Homology Region 2-Containing Protein Tyrosine Phosphatase-2 (SHP2)

**DOI:** 10.3390/molecules26040848

**Published:** 2021-02-05

**Authors:** Se Jeong Kwon, Dohee Ahn, Hyun-Mo Yang, Hyo Jin Kang, Sang J. Chung

**Affiliations:** 1 Department of Biopharmaceutical Convergence and School of Pharmacy, Sungkyunkwan University, Suwon 16419, Korea; sejung1110@naver.com (S.J.K.); ehgml94@naver.com (D.A.); 2 AbTis Co. Ltd., Suwon 16648, Korea; sweetlov79@gmail.com; 3 Chong Kun Dang Research Institute, CKD Pharmaceuticals, Gyeonggi-do 16995, Korea; hmyang@ckdpharm.com

**Keywords:** natural products, protein tyrosine phosphatase (PTP), src homology region 2-domain-containing phosphatase-1 (SHP-1), src homology region 2-containing protein tyrosine phosphatase-2 (SHP2), polyphyllin D, leukemia

## Abstract

Natural products have continued to offer tremendous opportunities for drug development, as they have long been used in traditional medicinal systems. SHP2 has served as an anticancer target. To identify novel SHP2 inhibitors with potential anticancer activity, we screened a library containing 658 natural products. Polyphyllin D was found to selectively inhibit SHP2 over SHP1, whereas two other identified compounds (echinocystic acid and oleanolic acid) demonstrated dual SHP1 and SHP2 inhibition. In a cell-based assay, polyphyllin D exhibited cytotoxicity in Jurkat cells, an acute lymphoma leukemia cell line, whereas the other two compounds were ineffective. Polyphyllin D also decreased the level of phosphorylated extracellular signal-regulated kinase (p-ERK), a proliferation marker in Jurkat cells. Furthermore, knockdown of protein tyrosine phosphatase (PTP)N6 (SHP1) or PTPN11 (SHP2) decreased p-ERK levels. However, concurrent knockdown of PTPN6 and PTPN11 in Jurkat cells recovered p-ERK levels. These results demonstrated that polyphyllin D has potential anticancer activity, which can be attributed to its selective inhibition of SHP2 over SHP1.

## 1. Introduction

Reversible protein tyrosine phosphorylation is critical in various cell signaling pathways. The degree of tyrosine phosphorylation is tightly regulated by the modulation of protein tyrosine kinases and protein tyrosine phosphatases (PTPs) [1]. The epithelial growth factor receptor (EGFR), a receptor tyrosine kinase, is a key regulator of various cellular processes, such as cell proliferation, differentiation, survival, and migration, which phosphorylates the tyrosine residues of proteins [2,3]. Overexpression or EGFR-activating mutations are closely related to the development and proliferation of many types of cancers, especially human leukemia, such as acute myeloid leukemia and acute lymphoblastic leukemia (ALL) [4].

PTPN11 (encoding SHP2) enhances EGFR signaling by dephosphorylating RasGAP, a negative regulator of the Ras pathway [5]. Moreover, SHP2 includes two Src homology 2 (SH2) domains located at the N-terminus (N-SH2) and C-terminus (C-SH2) of the PTP catalytic domain [6]. The N-SH2 domain negatively regulates PTP activity through intramolecular binding to the catalytic domain in the cellular environment. In tumors, growth factor receptor bound protein 2 (GRB2)-associated binding protein 1 (GAB1) activates SHP2 through its binding to N-SH2, thereby inactivating RasGAP, resulting in Ras activation [5,7]. This leads to tumorigenesis, growth, survival, and metastasis of cells via the activation of the Raf/MEK/ERK pathway [8]. In addition, conditional knockout experiments of SHP2 alleles have demonstrated that it is also essential for ErbB2 induced tumorigenesis [9]. The tyrosine phosphatase SHP2 inhibition and knockout in colorectal carcinoma HCT-116 cells promotes growth in vitro as well as in vivo, suggesting that the loss of SHP2 activity promotes the growth of HCT-116 cells [10]. Hence, inhibition of SHP2 may results in opposing scenarios. As shown in breast cancer model [9], its silencing blocks oncogene expression and tumorigenesis, while in a colorectal cancer model [10] loss of SHP2 activity promotes growth of colorectal carcinoma. Thus, it seems that the role of SHP2 is highly cell-type specific.

**Figure 1:**
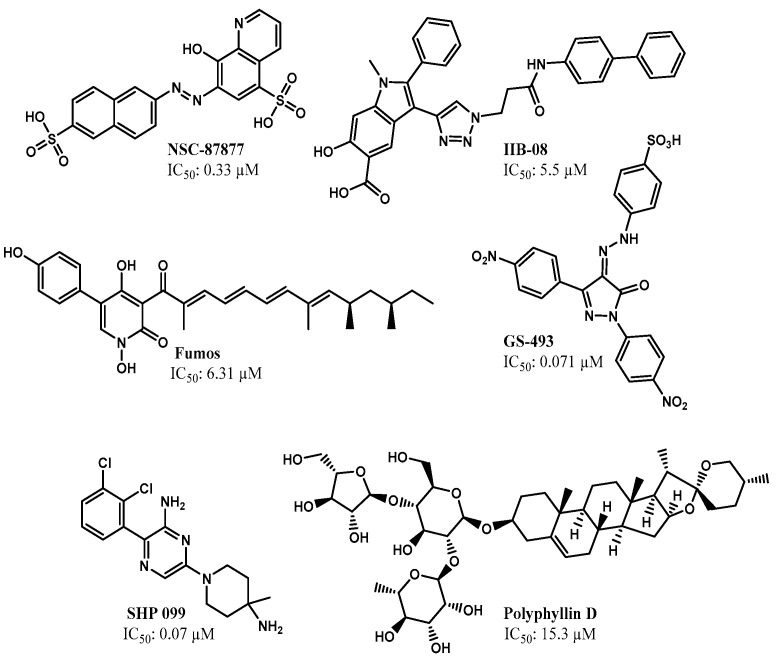
Chemical structures of representative SHP2 inhibitors. Fumos, NSC-87877, IIB-08, and GS-493 are active site inhibitors, whereas SHP099 and polyphyllin D are allosteric inhibitors.

Recently, the development of SHP2 inhibitors as potential antitumor agents has attracted increased attention. Four SHP2 allosteric inhibitors have already entered clinical trials for the treatment of solid tumors [11]. Many SHP2 inhibitors are reported to possess the anticancer activity (Figure 1) [12,13,14,15,16]. However, most exhibit low selectivity to SHP2 over the same class of PTPs or display poor cell permeability due to the presence of polar and ionic functional groups [17]. The PTP domain of SHP2 shares 75% sequence similarity with that of SHP1 and 34% with PTP1B [18,19]. However, SHP1 has demonstrated tumor-suppressor activity in leukemia and lymphomas, mainly due to the negative regulation of oncogenic JAK/STAT3 signaling [20]. Recently, SHP099 was reported as a potent allosteric SHP2 inhibitor (IC_50_ = 0.07 µM), with no inhibitory activity against SHP1 [12]. Its co-crystal structure with SHP2 reveals that SHP099 binds to a tunnel-like pocket comprising three domains (tunnel, latch, and groove) in SHP2 [21]. The progress in the recent years has spurred great interest in the development of allosteric SHP2 inhibitors.

This motivated us to investigate natural products with SHP2 selectivity as potential anticancer agents, which represents a continuation of our laboratory efforts to identify natural product-derived drugs for various diseases [22,23]. Herein, we screened 658 natural products against non-receptor-type PTPs to identity new SHP2 selective inhibitors. 

## 2. Results and Discussions

### 2.1. Expression, Purification, and Kinetic Characterization of SHP2

To identify new SHP2 anticancer natural products, we first performed cloning to express SHP2 protein and then purified it. PTPN11 (Gene ID: 5781) encoding human SHP2 (amino acids sequences 1 to 526) with several extra amino acids were cloned into the MBP-pET 28a (+) vector, which was successfully used to overexpress PTPs with enhanced solubility in an *Escherichia coli* expression system [24]. Information on amino acids for 15 non-receptor-type PTPs including SHP2 is summarized in Table S3 in the Supplementary Materials. SHP2 was overexpressed in RosettaTM (DE3) and purified using a metal affinity resin (Figure S1 in Supplementary Materials). The catalytic activity of the purified proteins was evaluated using DiFMUP as a fluorogenic substrate, which has been widely used to determine PTP activity [25]. The *k*_cat_, *K*_M_, and *k*_cat_/*K*_M_ values were determined to be 270 min^−1^, 70 μM, and 3.9 μM^−1^min^−1^, respectively, using the Lineweaver–Burk plot analysis (Table S1 in Supplementary Materials).

### 2.2. Identification of SHP2 Inhibitors from Natural Products

**Figure 2:**
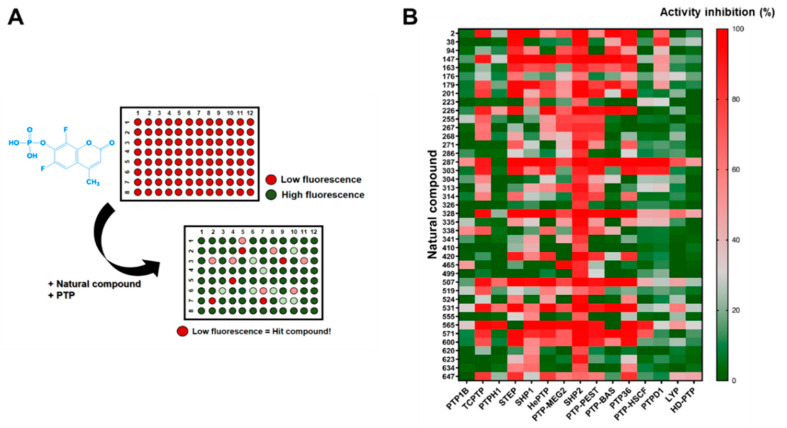
Screening of a library containing 658 natural compounds for selective SHP2 inhibitors. (**A**) The first round of screening employed the fluorogenic protein tyrosine phosphatase (PTP) substrate, DiFMUP (6,8-difuoro-4-methylumbiliferyl phosphate) in 96-well plates. DiFMUP (non-fluorescence) is dephosphorylated by PTP generating DiFMU, which is highly fluorescent (Ex/Em = 350/450 nm). Compounds resulting in low fluorescence were selected as potent SHP2 inhibitors. (**B**) Heatmap analysis of the natural compounds showing the inhibition of 15 PTPs by GraphPad Prism^®^.

Next, we established a screening strategy using a DiFMUP substrate to identify a natural product that inhibits SHP2 activity. Initially, we screened 658 compounds at 20 µM using a 96-well plate system (Figure 2A). In the first round of screening, 41 compounds inhibited SHP2 by more than 60%, which were then selected for further screening. The selected compounds were tested at 20 µM for SHP2 inhibition, and 14 additional non-receptor-type PTPs (PTP1B, TCPTP, PTPH1, STEP, SHP1, HePTP, PTP-MEG2, PTP-PEST, PTP-BAS, PTP36, PTP-HSCF, PTPD1, LYP, and HD-PTP) were used to investigate the inhibition selectivity. The results were visualized using a heat map analysis by GraphPad Prism (Figure 2B and Table S2 in Supplementary Materials).

**Figure 3:**
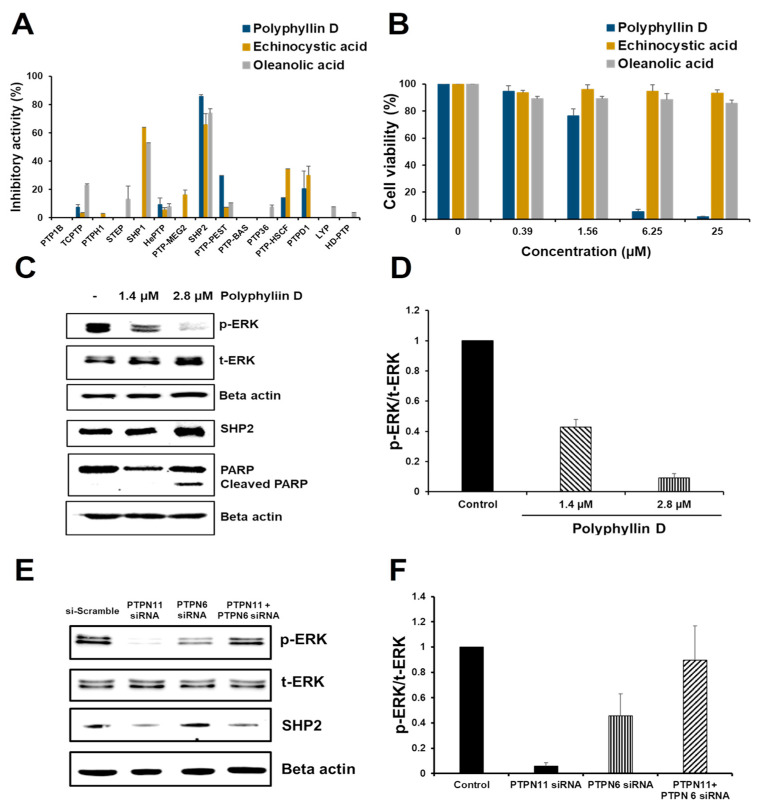
Validation of selective SHP2 inhibition. (**A**) Inhibitory activities of polyphyllin D, echinocystic acid, and oleanolic acid at 20 µM against representative non-receptor-type PTPs. (**B**) Cytotoxic activity of polyphyllin D, echinocystic acid, and oleanolic acid against Jurkat cells. The cells (3.5 × 10^5^ cells/mL) were seeded in a 96-well plate, incubated for 24 h, and then treated with different compounds (0.5% DMSO) at the appropriate concentrations (0.39, 1.56, 6.25, 25 µM) for 48 h. Cell viability was measured using the EZ-Cytox kit. (**C**) Western blot analysis showing the change in the level of p-ERK/t-ERK, and cleaved PARP from Jurkat cells. The cells were incubated with 1.4 and 2.8 μM of Polyphylin D. After 1 h, cells were lysed and Western blotting was performed for p-ERK and t-ERK, and after 24 h for SHP2 and cleaved PARP. (**D**) Quantification of p-ERK and t-ERK using ATTO image analysis software (CS analyzer 4). (**E**) Jurkat cells were transfected with PTPN11 and/or PTPN6 siRNAs, or scrambled siRNA as a control, and the expression of p-ERK was confirmed by the Western blot. (**F**) Quantification of total-ERK and phospho-ERK using the ATTO image analysis software. Results are expressed as the mean value ± the standard deviation of the mean value.

All tested compounds strongly inhibited the 15 PTPs, except for polyphyllin D, echinocystic acid, and oleanolic acid. Of these, polyphillin D presented the highest selectivity for SHP2 over the other PTPs. Echinocystic and oleanolic acids selectively inhibited SHP1 and SHP2 over the other PTPs tested (Figure 3A and Table S3 in Supplementary Materials). These three compounds were further tested for anticancer activity.

### 2.3. Effect of Polyphyllin D for the Proliferation and Signaling of Jurkat Cells

SHP2 is overexpressed in human leukemias compared to peripheral blood mononuclear cells (PBMC). Particularly, among various leukemia cell lines, Jurkat cells express the highest levels of SHP2 [26]. Therefore, Jurkat cells, an ALL cell line, were selected to evaluate the anticancer activity of the three selected compounds. Interestingly, only polyphyllin D strongly inhibited Jurkat cell viability, with an IC_50_ of 2.8 µM (Figure 3B), while the other compounds were ineffective.

SHP2 mediates the activation of the Ras/Raf/MEK/ERK pathway. To determine whether polyphyllin D suppressed SHP2-mediated Ras/Raf/MEK/ERK activation, Jurkat cells were treated with polyphyllin D for 1 h. Western blot analysis revealed that polyphillin D dose-dependently decreased p-ERK and increased cleaved PARP, an apoptotic marker, in Jurkat cells (Figure 3C,D). In addition, polyphyllin D did not change the level of mRNA and protein expression of SHP2. (Figure 3C and Figure S2 in Supplementary Materials). Considering that SHP2 activates the Ras/Raf/MEK/ERK pathway, these data indicate that polyphyllin D induced Jurkat cell apoptosis via SHP2 inhibition.

### 2.4. siRNA Knockdown Study of PTPN6 and PTPN11 in Jurkat Cells

PTPN11 is an established anticancer target, as evident from siRNA knockdown studies [27,28]. Notable results were found in the present study. Among the three natural product inhibitors selected, two SHP2 inhibitors, echinocystic and oleanolic acids, failed to induce Jurkat cell apoptosis. In addition, echinocystic acid and oleanolic acid did not change the level of p-ERK and cleaved PARP (Figure S3 in Supplementary Materials). This was further investigated via siRNA knockdown studies of PTPN6 and/or PTPN11, resulting in a significant change in the corresponding mRNA expression level (Figure S4 in Supplementary Materials). Knockdown of PTPN6 or PTPN11 alone significantly decreased p-ERK in Jurkat cells. Conversely, the double knockdown of both genes significantly recovered p-ERK levels compared to the knockdown of a single gene (Figure 3E,F). These results were strongly correlated with the observation that echinocystic and oleanolic acids, dual inhibitors of SHP1 and SHP2, did not inhibit the viability of Jurkat cells. The reason for the restoration of p-ERK by dual inhibition of SHP1 and SHP2 is not known. Decrease of ERK phosphorylation was greater with mRNA knockdown of SHP2 than that of SHP1, meaning that SHP2 is more responsible for ERK phosphorylation than SHP1 (Figure 3E). Considering the similarity of SHP1 and SHP2 in amino acid sequences as well as activation mechanism upon C-terminal phosphorylation [29] it is conjectured that SHP1 might compete with SHP2 in activation mechanism especially when SHP2 level is low in cells. However, the exact mechanism remains to be further studied.

Polyphyllin D is isolated from the traditional medicinal herb *Paris polyphylla*, and its extract is known to possess anticancer activity [30]. Polyphyllin D has been reported to elicit depolarization of the mitochondrial transmembrane potential, leading to H_2_O_2_ generation, release of cytochrome C, and apoptosis-inducing factors [31]. Furthermore, SHP2 was reported to dephosphorylate adenine nucleotide translocase 1 (ANT1) and subsequently control the mitochondrial permeability transition, thereby preventing the collapse of the mitochondrial membrane potential and the subsequent apoptotic process [32]. The current results, together with previous reports, suggest that polyphyllin D exerts anticancer activity through SHP2 inhibition, followed by collapse of the mitochondrial membrane potential, and subsequent Jurkat cell apoptosis.

### 2.5. Kinetic Study of Polyphyllin D for SHP2 Inhibition

**Figure 4:**
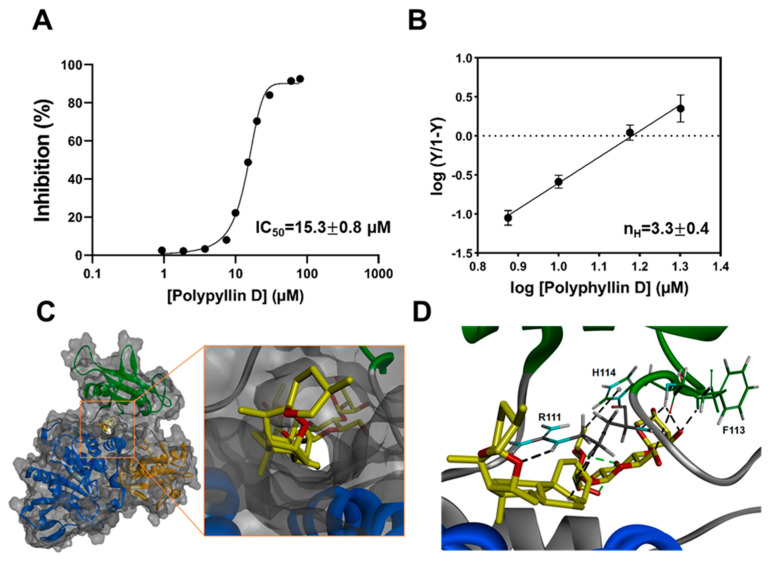
Biochemical evaluation of SHP2 inhibition by polyphyllin D. (**A**) Dose-dependent SHP2 inhibition by polyphyllin D (IC_50_ was 15.3 ± 0.8 µM). (**B**) Hill plot analysis. Hill coefficients (nH), i.e., the slopes of the plot, were 3.3 ± 0.4. (**C**) Docking analysis of polyphyllin D binding to SHP2 (PDB ID: 5EHR, orange, N-SH2; green, C-SH2; bleu, PTP domain). (**D**) Key interactions between polyphyllin D and SHP2 (R 111, F 113, H 114). Results are expressed as the mean value ± the standard deviation of the mean value.

To study the mechanism of SHP2 inhibition by polyphyllin D, we examined the inhibitory kinetics of polyphyllin D against SHP2. The progress curves of DiFMUP hydrolysis by SHP2 demonstrated linearity in the absence or presence of polyphyllin D, indicating that inhibitor binding is in fast equilibrium rather than slow-tight (Figure S5 in Supplementary Materials). The dose–response curve of polyphyllin D for SHP2 inhibition presented a sigmoidal shape, which implied an allosteric inhibition with an IC_50_ of 15.3 µM (Figure 4A).

Lineweaver–Burk or Dixon plots could not be used to determine K_i_ values, which suggests that polyphyllin D is a mixed inhibitor type, with multiple binding sites. The progress data were replotted using the Hill equation to determine the degree of cooperativity. Hill plot analysis revealed that the Hill coefficient (nH) was 3.3, indicating that polyphyllin D binds to SHP2 with positive cooperation (Figure 4B). Molecular docking predicted that the binding site of polyphyllin D onto SHP2 (PDB ID: 5EHR) would be the same as that of SHP099. Among the conformers generated by LigPrep, only the corresponding conformation was able to bind with an appropriate negative value (CDocker_Interaction_Energy = −16.6713 Kcal/mol). We predicted that the ligand would be oriented to the binding pose, as shown in Figure 4C,D. However, it is unclear whether the predicted site is the only binding site for polyphyllin D. The IC_50_ value of polyphyllin D for SHP2 inhibition was greater than that for cell growth inhibition. This may indicate that SHP2 is not the only target of polyphyllin D. Moreover, polyphyllin D is reported to inactivate the Wnt/β-catenin pathway by decreasing the amount of p-GSK-3β [33]. This combined mechanism would potentiate the anticancer effects of polyphyllin D in Jurkat cells.

## 3. Material and Methods

### 3.1. Materials

Primers were synthesized and DNA was sequenced by Bioneer (Daejeon, Korea) and Cosmogenetech (Seoul, Korea), respectively. Fluorogenic phosphatase substrate 6,8-difluoro-4-methylumbelliferyl phosphate (DiFMUP) was purchased from Life Technologies (San Jose, CA, USA). Phosphatase activity was assessed by recording the fluorescence intensities at Ex/Em = 355/460 nm using a VictorTM X4 multilabel plate reader (Perkin Elmer Korea, Daejeon, Korea). The natural compound library was purchased from Biopurify Phytochemicals Ltd. (Chengdu, China). T-cell ALL cells (Jurkat) were obtained from the Korean Cell Line Bank (Seoul, Korea) and maintained in RPMI-1640 (Gibco, New York, NY, USA) supplemented with 10% heat-inactivated fetal bovine serum (FBS; Gibco, New York, NY, USA) and penicillin/streptomycin (Gibco, New York, NY, USA).

### 3.2. Construction of SHP2 Overexpression Vectors

SHP2 was expressed as a fusion protein with an N-terminal maltose binding protein (MBP) tag using the MBP-pET 28a (+) vector (Merck Millipore, Darmstadt, Germany) to improve protein solubility. Human SHP2 genes were amplified via polymerase chain reaction (PCR) using appropriate primers and corresponding cDNAs as templates (Korea Human Gene Bank, KRIBB; Daejeon, Korea). The PCR products were inserted into the MBP-pET 28a (+) vector, which was digested with the corresponding restriction enzymes. The forward and reverse primers used were P1 and P2 for human SHP2 (P1 (NdeI site underlined): 5-GGAATTCCATATGACATCGCGGAGATGGTTT-3, P2 (XhoI site underlined): 5-CCGCTCGAGCTGTAGTGTTTCAATATAATGCTGG-3).

### 3.3. Expression and Purification of Recombinant SHP2

The resulting N-terminal MBP and C-terminal His6-tagged human SHP2 genes were transformed into *E. coli* RosettaTM (DE3) (Merck Millipore, Darmstadt, Germany). Recombinant SHP2 expression was induced by adding 1 mmol/L IPTG at 291 K for 16 h. The cells were harvested by centrifugation (3570× *g* at 277 K for 10 min) (1580R; Labogene, Daejeon, Korea), washed with buffer A (50 mmol/L Tris pH 7.5, 500 mmol/L NaCl, 5% glycerol, 0.05% 2-mercaptoethanol, and 1 mmol/L phenylmethylsulfonyl fluoride (PMSF), and lysed by ultrasonication. After centrifugation (29,820× *g* at 277 K for 30 min), the supernatant was incubated with a cobalt affinity resin (TALON^®^, Takara Korea, Seoul, Korea) on a rocker at 277 K for 1 h, and the resin was then washed with buffer A containing 1 mmol/L imidazole. SHP2 was eluted with buffer A supplemented with 100 mmol/L imidazole and stored at 193 K. Using a similar method, the other PTPs were prepared as previously described [23].

### 3.4. Kinetic Characterization of SHP2 Enzyme Activity

Enzymatic reactions were initiated by the addition of SHP2 (final concentration of 10 nM) to a DiFMUP solution at a series of concentrations (final concentrations of 800, 400, 200, 100, 50, 25, 12.5, and 6.25 µM) in 100 μL of reaction buffer (20 mM bis-Tris pH 6.0, 150 mM NaCl, 2.5 mM dithiothreitol, 0.01% Triton X-100, and 2.5 mM EDTA) in a 96-well plate. The change in fluorescence intensity was measured at Ex/Em = 355/460 nm using a VictorTM X4 multilabel plate reader, whereas the *K*_M_ and *V*_max_ values were obtained using Hyper32 software (University of Liverpool, Liverpool, UK). The value was calculated from *V*_max_ and the enzyme concentration used.

### 3.5. Screening of Natural Compound Library for SHP2 Inhibition

Enzymatic reactions were initiated by the addition of SHP2 (final concentration of 10 nM) to a solution of each of the 658 phytochemical compounds (final concentration of 20 µM) in reaction buffer (20 mM bis-tris pH 6.0, 150 mM NaCl, 2.5 mM dithiothreitol, and 0.01% Triton X-100) containing DiFMUP (final concentration of 140 µM). Changes in the fluorescence intensities were measured continuously for 10 min at Ex/Em = 355/460 nm on a VictorTM X4 multilabel plate reader. Enzyme inhibition was estimated by comparing the reaction velocity in the presence of each compound with that in the absence of any compound. Inhibition of the other PTPs was measured in the same way.

### 3.6. Assessment of Antitumor Activity

Jurkat cells were maintained in RPMI-1640 supplemented with 10% FBS, whereas the cell media were supplemented with penicillin (100 U/mL) and streptomycin (100 µg/mL) in 5% CO_2_ at 310 K. The cells (3.5 × 10^5^ cells/mL) were seeded in a 96-well plate, incubated for 24 h, and then treated with different compounds (0.5% DMSO) at the appropriate concentrations (0.39, 1.56, 6.25, 25 µM) for 48 h. Cell viability was measured using the EZ-Cytox kit (Dogen, Seoul, Korea) the kit reagent was added to each well and the mixture was incubated for 3.5 h under standard culture conditions. Next, the absorbance of the treated and untreated samples was measured at 450 nm using a VictorTM X4 multilabel plate reader. The IC_50_ value represents the inhibitory concentration that causes 50% growth inhibition of cells.

### 3.7. Western Blot Analysis

The cell lysates in RIPA buffer (Sigma-Aldrich Korea, Yongin, Korea) were separated via sodium dodecyl sulfate polyacrylamide gel electrophoresis (SDS-PAGE), transferred to PVDF membranes (Immobilon^®^, Millipore, Darmstadt, Germany), and then blocked in 5% non-fat skim milk in TBS-T (10 mM Tris-HCl pH 7.5, 150 mM NaCl, and 0.05% Tween-20) for 1.5 h at room temperature. The membranes were subsequently probed with the following primary antibodies (Cell Signaling Technology, Danvers, MA, USA): PARP (#9542), SHP-2 (#3752), phospho-p44/42 MAPK (#9101), p44/42 MAPK (#4695), and anti-beta-actin N-term (LF-PA0207; AbFrontier, Seoul, Korea) at 277 K overnight. Thereafter, the primary antibodies were bound to horseradish peroxidase (HRP)-conjugated secondary antibodies (goat anti-rabbit IgG-HRP, sc-2004, Santa Cruz Biotechnology, Dallas, TX, USA). The amount of target protein was measured using enhanced chemiluminescence (GE Healthcare, Beirut, Lebanon).

### 3.8. RNA Interference and Quantitative Real Time-Polymerase Chain Reaction (qRT-PCR)

Knockdown of PTPN6 and PTPN11 in Jurkat cells was performed using small interfering RNAs (siRNAs, Genolution Pharmaceuticals Inc., Seoul, South Korea). siRNA(s) at 20 nM final concentration was transfected into Jurkat cells using the Neon^®^ transfection system (Invitrogen, Carlsbad, CA, USA). For the efficient knockdown of the target genes, a combination of three siRNAs was employed, and the sequences (5’-sense-3’/5’-antisense-3’) are GCA AUG ACG GCA AGU CUA AUU/UUA GAC UUG CCG UCA UUG CUU; GAG UGA UUG UCA UGA CAA CUU/GUU GUC AUG ACA AUC ACU CUU; GAG GAA AGG GCA CGA AUA UUU/AUA UUC GUG CCC UUU CCU CUU for PTPN11 (Gene ID: 5781), and GAG UGU UGG AAC UGA ACA AUU/UUG UUC AGU UCC AAC ACU CUU; GAG UUU GAG AGU UUG CAG AUU/UCU GCA AAC UCU CAA ACU CUU; GCA AGA ACC GCU ACA AGA AUU/UUC UUG UAG CGG UUC UUG CUU for PTPN6 (Gene ID: 5777). For measuring mRNA or protein expression, cells were harvested 24 h for qRT PCR or 48 h for Western blot analysis after siRNA transfection. The total RNA was isolated from the Jurkat cells using RNeasy Mini kit (Qiagen, Valencia, CA, USA) and treated with DNase (Qiagen) to remove genomic DNA. The total RNA (1 μg) was used to synthesize cDNA with the High Capacity Reverse Transcription kit (Applied Biosystems, Foster City, CA, USA). RT PCR was performed on a CFX Connect Real-Time PCR Detection System (Bio-Rad, Hercules, CA, USA) using SsoAdvanced Universal SYBR green supermix (Bio-Rad) according to the manufacturer’s instructions. The gene expression levels of PTPN11 and PTPN6 were measured using forward/reverse primers (GCC TGC AAA ACA CGG TGA AT/AGC GTA TAG TCA TGA GCG GC for PTPN11 and AAC AAG CAG GAG TCC GA/ATG TTA CTG TCC CGT CCC TG for PTPN6) and normalized to the level of the control gene, RPLPO (forward/reverse primers: CAG ATT GGC TAC CCA ACT GTT/GGG AAG GTG TAA TCC GTC TCC).

### 3.9. Docking Study of Polyphyllin D into SHP2

The crystal structure of SHP2 (PDB ID: 5EHR) was selected from the RCSB Protein Data Bank as a template structure for the docking study. The water molecules in the crystal structure were removed, and the binding site was defined by creating a sphere with a radius of 17 Å in the region where the original binding ligand was situated. The docking studies were performed using the CDOCKER module of the Discovery Studio 4.1 software and the results were scored using the CDOCKER Interaction Energy function. The structure of the ligand (Polyphyllin D, CAS No. 50773-41-6) was drawn on a ChemDraw software (ChemBioDraw Ultra 12.0, Cambridgesoft, Cambridge, MA, USA) to convert it into a mol file, followed by the generation of a three-dimensional structure using LigPrep (Schrödinger Release 2018-3: LigPrep, Schrödinger, LLC, New York, NY, 2018). The resulting ligand poses were used as the input ligand for the CDOCKER module.

### 3.10. Statistical Data Analysis

The IC_50_ values were determined by plotting the relative cell growth versus inhibitor concentrations using the KaleidaGraph software (version 4.0, Synergy Software, Reading, PA, USA). GraphPad Prism (version 7, GraphPad Software, Inc., La Jolla, CA, USA) was used to prepare the heat maps.

## 4. Conclusions

We identified polyphyllin D as a potent selective SHP2 inhibitor. Treatment with polyphyllin D decreased p-ERK levels and increased cleaved PARP levels in Jurkat cells, leading to apoptotic cell death. The knockdown of PTPN6 or PTPN11 mRNA decreased p-ERK; however, concurrent mRNA knockdown of PTPN6 and PTPN11 by siRNA increased the level of p-ERK. Furthermore, echinocystic and oleanolic acids presented dual inhibition of SHP1 and SHP2, but they did not inhibit the viability of Jurkat cells. These results imply that selective inhibition of SHP2 over SHP1 is critical for anticancer activity. Enzyme kinetics revealed that polyphyllin D is a mixed SHP2 inhibitor, with an IC_50_ value of 15.3 µM.

## Data Availability

Data is contained within the article or Supplementary Materials.

## Supplementary Materials

The following are available online, Figure S1: Purification and kinetic evaluation of SHP2, Figure S2: qRT-PCR analysis of PTPN11 mRNA expression in the presence of polyphyllin D without siRNA, Figure S3: Validation of selective SHP2 inhibition, Figure S4: qRT-PCR analysis of PTPN11, PTPN6 knockdown using siRNA, Figure S5: SHP2 inhibition by polyphyllin D, Table S1: Kinetic constants for DiFMUP hydrolysis by SHP2, Table S2: Details of selected candidates obtained from a 658-membered natural product library, Table S3: Selectivity profile of polyphyllin D compared with various non-receptor-type PTPs.
