# Supplementary material for: Polyphyllin D Shows Anticancer Effect through a Selective Inhibition of Src Homology Region 2-Containing Protein Tyrosine Phosphatase-2 (SHP2)
Source: Molecules. 2021 Feb 5;26(4):848. doi: 10.3390/molecules26040848 (PMC7915588; doi:10.3390/molecules26040848)
Supplement: Supplementary file 1 [file molecules-26-00848-s001.pdf]

Supplementary Materials

Polyphyllin D Shows Anticancer Effect Through a Selective Inhibition of Src Homology Region 2-Containing Protein Tyrosine Phosphatase-2 (SHP2)

Se Jeong Kwon[1,2], Dohee Ahn[1], Hyun-Mo Yang[3], Hyo Jin Kang[2], Sang J. Chung[1,2*]

[1] School of Pharmacy, Sungkyunkwan University, Suwon 16419, Republic of Korea; sejung1110@naver.com (S. J. K.); ehgml94@naver.com (D. A.)
[2] AbTis Co. Ltd., Suwon, Republic of Korea; sweetlov79@gmail.com (H. J. K.)
[3] Chong Kun Dang Research Institute, CKD Pharmaceuticals, Republic of Korea; hmyang@ckdpharm.com (H.-M. Y.)
* Correspondence: sjchung@skku.edu (S. J. C.); Tel.: +82-31-290-7703

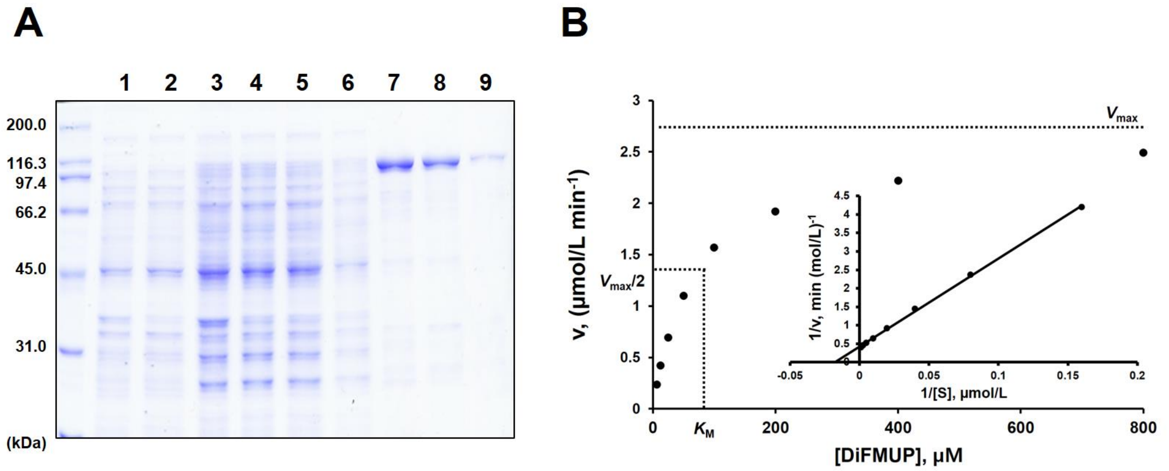

Figure S1. Purification and kinetic evaluation of SHP2. (A) SDS-PAGE analysis of SHP2. SHP2 (M.W. = 103.5 kDa) was overexpressed in *Escherichia coli* and purified via cobalt affinity chromatography (line 1: uninduced total cell lysate; line 2: uninduced soluble cell lysate; line 3: total cell lysate; line 4: soluble cell lysate; line 5: sample passed through the affinity column; lines 6 and 7: washed with a 1 mM imidazole buffer; lines 8 and 9: elution with a 100 mM imidazole buffer). (B) Kinetic analysis of SHP2 activity using DiFMUP as a substrate (K_M = 70 μM) by means of a Michaelis–Menten plot. A Lineweaver–Burk plot analysis is shown in the inset.

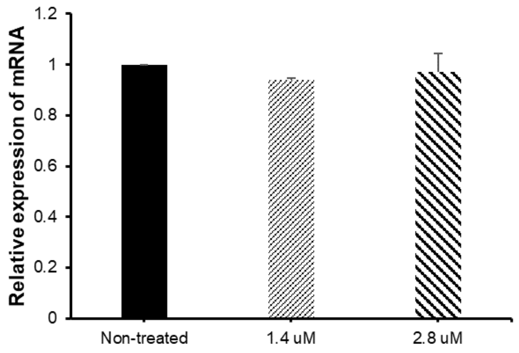

Figure S2. qRT-PCR analysis of PTPN11 mRNA expression in the presence of polyphyllin D without siRNA. The cells were treated with polyphyllin D (1.4, 2.8 μM) for 24 h. The mRNA expression of PTPN11 was analyzed after polyphyllin D treatment. Results are expressed as the mean value ± the standard deviation of the mean value.

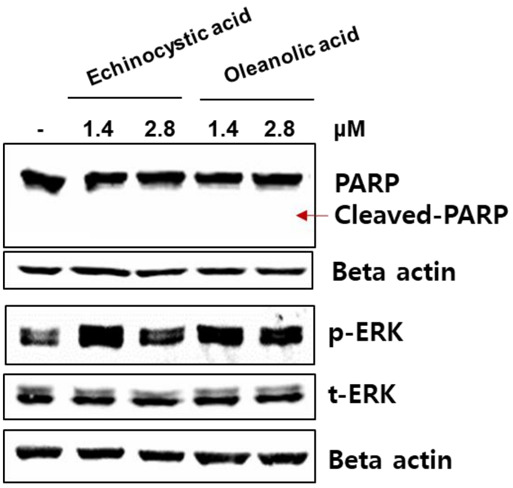

Figure S3. Validation of selective SHP2 inhibition. Western blot of p-ERK, t-ERK, and cleaved PARP from Jurkat cells treated with echinocystic acid and oleanolic acid. The cells were treated with echinocystic acid and oleanolic acid (1.4 and 2.8 μM) for 1 h and 24 h, then the cells were lysed for performing western blotting of p-ERK/t-ERK and cleaved PARP.

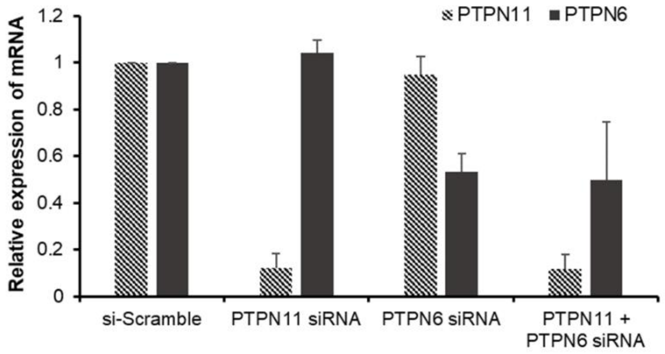

Figure S4. qRT-PCR analysis of PTPN11, PTPN6 knockdown using siRNA. Jurkat cells were transfected with PTPN11, PTPN6 siRNAs or scrambled siRNA as a control. The mRNA expression of PTPN11 and PTPN6 was analyzed after siRNA treatment. Results are expressed as the mean value ± the standard deviation of the mean value.

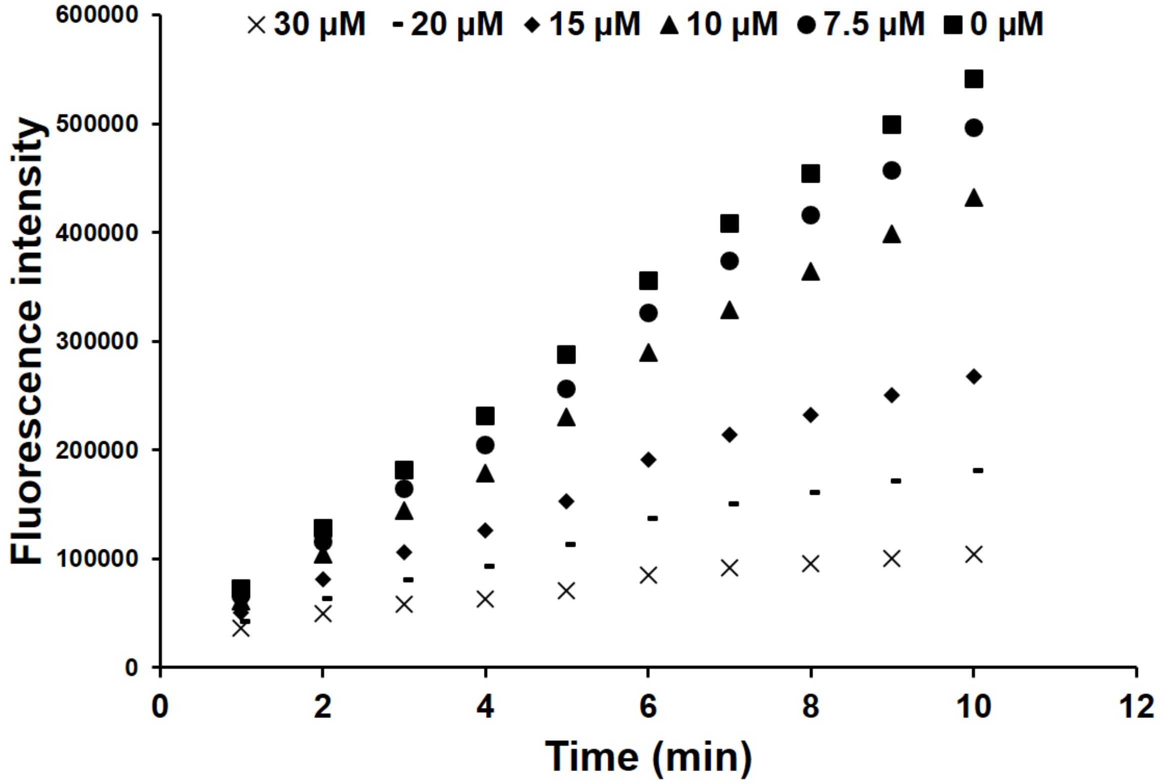

Figure S5. SHP2 inhibition by polyphyllin D. Curves show DiFMUP hydrolysis by SHP2 in the presence of polyphyllin D: 30 μM (×), 20 μM (-), 15 μM (♦), 10 μM (▲), 7.5 μM (●), and 0 μM (■).

Table S1. Kinetic constants for DiFMUP hydrolysis by SHP2.

	[E] (nM)	K_M (μM)	V_{max} (μMmin^{-1})	k_{cat} (min^{-1})	k_{cat}/K_M (μM^{-1} min^{-1})
SHP2	10	70	2.7	270	3.9

Table S2. Details of selected candidates obtained from a 658-membered natural product library.

	Compound number	Chemical name	CAS number	M.W(g/mol)
1	2	1,2,3,4,6-o-Pentagalloylglucose	14937-32-7	940.7
2	38	Acetate gossypol	12542-36-8	578.6
3	94	Baicalein	491-67-8	270.2
4	147	Chebulinic acid	18942-26-2	956.7
5	163	Corilagin	23094-69-1	634.5
6	176	Curcumin	458-37-7	368.4
7	179	Cyanidin chloride	528-58-5	322.7
8	201	Demethylzeylasteral	107316-88-1	480.6
9	223	Echinocystic acid	510-30-5	472.7
10	226	Elllagic acid	476-66-4	302.2
11	255	Fisetin	528-48-3	286.2
12	267	Gallic acid ethyl ester	831-61-8	198.2
13	268	Gallocatechin gallate (GCG)	4233-96-9	458.4
14	271	Gambogic acid	2752-65-0	628.8
15	286	Ginkgetin	481-46-9	566.5
16	287	Ginkgolic acid C13-0	20261-38-5	320.5
17	303	Gossypol	303-45-7	518.6
18	304	Gracillin	19083-00-2	885.0
19	313	Hederagenin	465-99-6	472.7
20	314	Nepetin	520-11-6	316.3
21	326	Roburic acid	6812-81-3	440.7
22	328	Hypericin	548-04-9	504.4
23	335	Neogambogic acid	93772-31-7	646.8
24	338	Isoanhydroicaritin	28610-30-2	368.4
25	341	Isoginkgetin	548-19-6	566.5
26	410	Maslinic acid	4373-41-5	472.7

27	420	Momordin Ic	96990-18-0	764.9
28	465	Pachymic acid	29070-92-6	528.8
29	499	Polyphyllin I; Polyphyllin D	50773-41-6	855.0
30	507	Protopseudohypericin	54328-09-5	522.5
31	519	Quercetin	117-39-5	302.2
32	524	Ranaconitine	1360-76-5	600.7
33	531	Rhynchophylline	76-66-4	384.5
34	555	Sciadopitysin	521-34-6	580.5
35	565	Sennoside A	81-27-6	862.7
36	571	Shikonin	54952-43-1	288.3
37	600	Tanshinone I	568-73-0	276.3
38	620	Oleanolic acid	508-02-1	456.7
39	623	Tormentic acid	13850-16-3	488.7
40	634	Ursolic acid	77-52-1	456.7
41	647	Wedelolactone	524-12-9	314.2

Table S3. Selectivity profile of polyphyllin D compared with various non-receptor-type PTPs.

No	Gene (Protein)	Gene ID	Amino acid sequence in recombinant PTPs	Inhibition by 20 μM polyphyllin D (%)
1	PTPN1 (PTP1B)	5770	1-299	0
2	PTPN2 (TCPTP)	5771	1-387	7.5
3	PTPN3 (PTPH1)	5774	403-913	0
4	PTPN5 (STEP)	84867	313-565	0
5	PTPN6 (SHP1)	5777	1-595	0.1
6	PTPN7 (HePTP)	5778	1-360	9.2
7	PTPN9 (PTP-MEG2)	5780	277-582	0
8	PTPN11 (SHP2)	5781	1-526	85.6
9	PTPN12 (PTP-PEST)	5782	1-334	29.9
10	PTPN13 (PTP-BAS)	5783	2095-2490	0
11	PTPN14 (PTP36)	5784	908-1187	0
12	PTPN18 (PTP-HSCF)	26469	1-300	14.2
13	PTPN21 (PTPD1)	11099	839-1174	20.5
14	PTPN22 (LYP)	26191	1-326	0.1
15	PTPN23 (HD-PTP)	25930	1179-1463	0
